# Polishing of AISI 304 SS by Electrolytic Plasma in Aqueous Urea Solution: Effect on Surface Modification and Corrosion Resistance

**DOI:** 10.3390/ma18163786

**Published:** 2025-08-12

**Authors:** Hugo Pérez-Durán, Francisco Martínez-Baltodano, Gregorio Vargas-Gutiérrez

**Affiliations:** Centro de Investigación y de Estudios Avanzados del Instituto Politécnico Nacional, Unidad Saltillo, Ramos Arizpe 25900, Mexico; hugopd23@gmail.com (H.P.-D.); fco.mtzbaltodano@cinvestav.edu.mx (F.M.-B.)

**Keywords:** surface treatment, plasma treatment of materials, 304 stainless steel, electropolishing, complex oxides

## Abstract

Plasma Electrolytic Polishing (PEP) is an advanced anodic process that enhances stainless steel surfaces through controlled electrochemical dissolution and plasma-mediated modification. This study demonstrates that PEP treatment of AISI 304 SS at 300 V in aqueous urea solution (3.0 wt.%)/NH_4_NO_3_ (0.25 wt.%) achieves remarkable improvements: surface roughness decreases by 54.6% (from 0.197 ± 0.023 μm to 0.0895 ± 0.0205 μm) with minimal mass loss (0.0035 g·cm^−2^) in just 20 min. Tafel analysis showed a 99% reduction in corrosion rate (0.00497 mm yr^−1^) compared to untreated AISI 304 SS (0.094 mm yr^−1^). Cyclic Potentiodynamic Polarization (CPDP) measurements indicated superior pitting resistance (E_pit_ = +0.423 vs. +0.486 V for PEP processing). XPS analysis elucidates the underlying mechanisms, showing a 91% increase in the Cr/Fe ratio (0.44 to 0.84) and complete transformation of surface oxides to protective Cr_2_O_3_ (57.34 wt.%) and Fe_3_O_4_ (55.88 wt.%), which collectively explain the enhanced corrosion resistance.

## 1. Introduction

Surface polishing of stainless steel is a crucial process for applications requiring high corrosion resistance and low surface roughness [1]. Among the available techniques, conventional electropolishing (EP) and Plasma Electrolytic Polishing (PEP) stand out for their ability to produce smooth surfaces through electrochemical processes, although they differ significantly in their implementation, efficiency, and environmental impact [2].

EP employs electrolytes based on acid solutions [3], ionic liquids (ILs) [4], and deep eutectic solvents (DESs) [5]. However, acid solutions present significant limitations, primarily due to the use of highly toxic electrolytes that generate environmentally harmful residues and pose health risks to operators. Conversely, ILs and DES are expensive, restricting their industrial application. EP typically operates under low-voltage conditions, generally between 6 and 18 V, with current densities ranging from 5 to 50 A cm^−2^. Its main mechanism involves the formation of a viscous layer on the anode, thinner over micro peaks compared to valleys, which favors the preferential removal of surface irregularities [6].

In contrast to conventional methods, PEP represents an innovative technological alternative characterized by using diluted and less toxic solutions, such as ammonium sulfate or urea mixed with nitrates [7]. These compounds have gained significant attention as they offer safer, more eco-friendly, and cost-effective alternatives to highly corrosive acid-based electrolytes or expensive ionic liquids used in conventional EP. Beyond simply enhancing electrical conductivity, these specific chemicals play crucial roles in facilitating the stable formation of the plasma sheath and influencing the electrochemical reactions on the metal surface. While other substances like carbonates, organic acids, or chlorides could be explored, these salts are typically preferred in PEP due to their favorable balance of safety, cost, and effectiveness in promoting controlled material removal and passivation without common issues such as severe pitting corrosion, often associated with halides. Unlike traditional electropolishing, PEP operates at significantly higher voltages (220–450 V), inducing the formation of a plasma state region around the anode. Under these conditions, the process combines thermal and electrochemical mechanisms, where the heat generated by Joule’s effect concentrates on topographical irregularities, promoting their localized dissolution in a highly selective manner, without the need for aggressive chemical reactions [8].

Various studies have demonstrated the applicability of PEP on different types of steel under specific conditions. For example, Zatkalíková et al. (2022) [2] reported treating AISI 316L steel using a 6 wt. % ammonium sulfate solution, applying 260 V and 68 °C for 3 min, with promising results for biomedical applications such as implants and surgical instruments. Complementarily, Sushil et al. (2024) [9] explored concentrations between 2 and 4% (*w*/*v*) of the same electrolyte on AISI 304 steel, operating between 200 and 400 V at temperatures of 70 to 90 °C for 1 to 3 min, aiming for high-precision industrial finishes. Similarly, Ji et al. (2024) [10] used 3 wt. % of ammonium sulfate to electropolish AISI 316L steel over a voltage range of 100 to 500 V and temperatures between 70 and 90 °C for 10 min, demonstrating the process’s flexibility for various applications. Other studies, such as that by Philipp Wahl et al. (2024) [11], reported the use of 5% (*w*/*v*) ammonium sulfate on the same substrate (AISI 316L), operating between 280 and 340 V at 70 °C for 10 to 20 min, achieving uniform and stable finishes. In more specific conditions, Sushil et al. (2025) [12] explored the treatment of AISI 304 steel using a 2% (*w*/*v*) ammonium sulfate solution at 350 V and 75 °C for 2 min, with the aim of optimizing electropolishing for components requiring low friction. Additionally, this study demonstrated the effectiveness of PEP on Inconel 718, a superalloy used in high-temperature environments, showing the versatility of the method for materials with high thermal demands. Finally, Wang et al. (2025) [13] evaluated a two-stage treatment for martensitic stainless steel 17-4PH, using a mixture of ammonium sulfate (2–5 wt. %) with a notable modification: the addition of sulfuric acid to adjust the pH between 3.1 and 3.2. The process involved an initial stage at 75 °C for 10 min, followed by a second phase at 90 °C for 20 min. The main goal was to study the application of PEP in surgical tools and fluid conduction systems. These studies demonstrated that PEP with ammonium sulfate is a versatile and effective technique to improve the surfaces of stainless steels (AISI 304, 316L, 17-4PH) for use in biomedical applications and superalloys like Inconel 718, especially for components operating under high-temperature conditions.

Despite these advances, most investigations on PEP have primarily focused on surface characteristics such as gloss or roughness, with little exploration of its corrosion performance, especially under conditions that simulate marine environments. Addressing this gap is one of the main objectives of the present study. Additionally, another specific goal is to analyze PEP applied to AISI 304 steel using an aqueous urea solution as the electrolyte. A key advantage of this approach is the stability of the electrolyte, as it is not consumed during the process, allowing for reuse. This helps to reduce operational costs and the environmental impact of the procedure. Although Hoar et al. (2017) [14] studied mixtures of urea and ammonium chloride for nickel electropolishing and Rotty et al. (2019) [15] examined the use of rielina (a urea–choline chloride mixture) to enhance the corrosion resistance of AISI 316L steel, to date, there are no reports on using aqueous urea solutions as electrolytes in PEP processes. This notable gap in the scientific literature motivated the current work, which introduces and evaluates an innovative electrochemical system based on an aqueous urea solution with a small addition of ammonium nitrate. The study focuses on three key aspects: the characterization of the corrosion resistance properties of the finished surface, the analysis of the mechanisms and phases involved, and the identification of crystalline compounds formed on the surface using XPS. The use of aqueous urea solutions represents a significant contribution to the sustainability of the process, as it reduces both operational costs and waste generation, aligning with current principles of environmental efficiency and a circular economy.

## 2. Materials and Methods

### 2.1. Plasma Electrolytic Polishing (PEP)

To carry out the PEP (Plasma Electrolytic Polishing) process, two stainless steel electrodes were used: a square cathode with an area of 35 cm^2^ and a circular anode with an area of 5.73 cm^2^. These substrates were metallographically prepared and polished using sandpaper with grit ranging from 80 to 1000, until achieving a mirror-like finish. The chemical composition (wt. %) of the electrodes consisted of 0.04 C, 0.005 S, 0.052 P, 0.5 Si, 1.36 Mn, 0.138% Mo, 7.7 Ni, 18.12 Cr, 68.2 Fe, and 3.88 other elements. Both electrodes were submerged inside a tempered glass container of 1 L capacity, into which 500 mL of the aqueous urea solution (CON_2_H_4_, 3.0 wt. %) (Fagalab**^®^**, Mocorito, SI, Mexico, 99.6%) was added. To increase the conductivity of the electrolyte, 0.25 wt. % of ammonium nitrate (Jalmek**^®^**, San Nicolas de los Garza, NL, Mexico, 95%) was also added. The electrodes were connected to a 6 kW power supply operating in an anodic mode. The PEP was performed in two stages. In stage 1, material removal was carried out at 140 V and 1.4 A cm**^−^**^2^. In the second stage, mirror polishing was performed for 10 min at 330 V, with a current density between 0.75 and 1.01 A cm**^−^**^2^. Finally, after treatment, samples were water-rinsed and ultrasonically cleaned in ethanol for 5 min.

### 2.2. Surface Characterization

The treated surfaces underwent extensive characterization to evaluate their modified properties: the surface chemical composition was analyzed by X-ray Photoelectron Spectroscopy (XPS) with a Thermo Scientific k-AlphaTM spectrophotometer with a camera at 6.5 × 10^−8^ mbar, Al-kα source, 400 μm analysis radius (AdvanceTM, Karlsruhe, Germany); surface morphology and roughness were evaluated using a KeyenceTM optical microscope (model VHX 6000, Keyence Corporation, Osaka, Japan)) for high-performance imaging; and resolution and analysis of three-dimensional roughness were carried out (3D scans of areas from 3 × 3 μm at 1000× to calculate $R_{q}$ and estimate the real area with prophylometry functions and a geometric model with L = 3 μm, applying Equation (1)) (Adapted from [16]). Additionally, the mass loss of the samples was evaluated. To do this, the samples were weighed with an OHAUS^®^ precision analytical balance (model DV214C, Long Branch, NJ, USA), with an accuracy of ±0.1 mg before and after treatment and exposure to corrosion.

(1) $$A_{real}=A_{geom}\sqrt{1+\left(\frac{4\pi \cdot R_{q}^{2}}{L^{2}}\right)}$$

### 2.3. Corrosion Resistance

To evaluate the corrosion resistance, the Cyclic Potentiodynamic Polarization (CPP) test was used. A three-electrode electrochemical cell was used (with platinum as a counter electrode, Ag/AgCl as a reference electrode, and an AISI 304 SS disk as a working electrode). The equipment used was a multichannel PARSTAT^®^ potentiostat, controlled by Versa Studio^®^ software v. 2.62.2. Deaeration with N_2_ was performed for 1 h to eliminate the oxygen present in the cell; then the open-circuit potential test was applied for 50 min. The CPP test was carried out at a scan rate of 0.167 mV s^−1^ for untreated SS and SS treated with PEP at 300 V, considering the ASTM G61-86 standard [17] and using simulated seawater as an electrolyte following the ISO 11130:2017 standard [18,19], the composition of which (in wt.%) was NaCl (58.46%), Na_2_SO_4_ (9.7%), MgCl_2_·6H_2_O (26.48%), CaCl_2_ (2.76%), SrCl_2_·6H_2_O (0.10%), KCl (1.66%), NaHCO_3_ (0.48%), KBr (0.24%), H_3_BO_3_ (0.06%), and NaF (0.01%). From the CPP curves obtained, the Tafel slopes of the anodic and cathodic curves were determined using EC-Lab^®^ software v. 10.19. With the calculated electrochemical corrosion parameters, the corrosion rate (*CR*) in mm per year was determined according to Equation (2).

(2) $$CR=k_{1}\cdot i_{corr}\cdot EW\cdot \rho^{-1}$$

where *k*_1_ is the corrosion constant, 3.27 × 10^−3^ mm g/μA cm yr; *i_corr_* is the corrosion current density, given in μA cm^−2^; *ρ* is the density (7.98 g cm^−3^); and *EW* is the SS equivalent weight, 24.27 eq.

The corrosion rate (*CR*) calculation from polarization resistance (*R_p_*) follows the ASTM G102 standard [20] procedure. This method employs linear polarization resistance (LPR) measurements, where a minor potential perturbation (Δ*E* = ±20 mV) is applied to the open-circuit corrosion potential (*E_corr_*) while monitoring the resulting current response (*i_med_*). The restricted potential window ensures system linearity while minimizing disturbance to the electrochemical equilibrium. The polarization resistance is derived using *R_p_* = Δ*E*/*I_med_* from this measurement. Subsequently, the Stern–Geary equation converts this R_p_ value to corrosion current density (*i_corr_*), which is then used to determine the corrosion rate through standard conversion factors. Then, the Stern–Geary constant (Equation (7)) is calculated using the anodic (*β_a_*) and cathodic (*β_c_*) Tafel slopes, which are calculated from Equations (3) and (4).

(3) $$y_{a}=\beta_{a}\cdot x+b_{a}$$

(4) $$y_{c}=\beta_{c}\cdot x+b_{c}$$

(5) $$B=\beta_{a}\cdot \beta_{c}\;\cdot {\left[2.303\cdot \left(\beta_{a}+\beta_{c}\right)\right]}^{-1}$$

Here, using *B* (Equation (5)) and *R_p_*, the corrosion current density (Equation (6)) is obtained, ensuring consistent units (*B* in V; *R_p_* in Ω·cm^2^). Finally, the corrosion rate (*CR*) is calculated by applying Equation (2).

(6) $$i_{corr}=B\cdot R_{p}^{-1}$$

Pitting corrosion resistance (*R_pit_*) was evaluated using Equation (7), where *E_corr_* is the steady-state corrosion potential and *E_pit_* is the pitting potential in V [21].

(7) $$R_{pit}=\left|E_{corr}-E_{pit}\right|$$

## 3. Results

### 3.1. Current Density/Potential Relationship for the PEP Process

**Figure 1:**
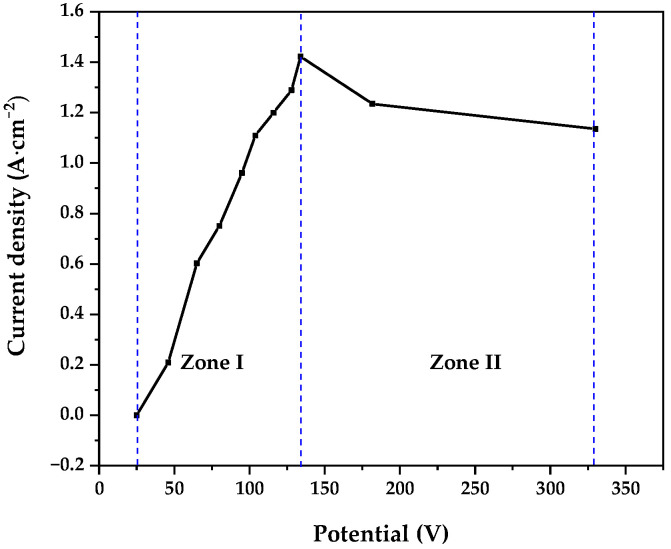
Characteristic voltage vs. current density behavior during PEP. For this treatment, an aqueous solution containing 3.0 wt. % urea and 0.25 wt. % NH_4_NO_3_ was used.

To achieve electrodissolution under plasma electrolytic (PEP) conditions, it is crucial to establish the potential and current density that enable plasma generation at the anode. Figure 1 illustrates the potential–current density relationship for the electrolyte used: an aqueous solution of 3.0 wt. % urea and 0.25 wt. % NH_4_NO_3_ by weight. Figure 1 shows two main zones:

- Zone 1: In this phase, a vapor sheath forms around the anode, and material removal begins. A maximum current density of 1.4 A cm^−2^ was reached at 140 V.
- Zone 2: At potentials higher than 140 V, the current density decreased, leading to this second zone.

This behavior has previously been reported by Kalenchukova et al. [22]. As the potential increases and the vapor sheath grows, dielectric breakdown of the sheath occurs, initiating the first electrical discharges. Plasma electrolytic conditions were achieved in the range of 250 to 340 V. Similar observations have been documented by Roy et al. [23] and by Yerokhin et al. [24].

For material removal from the anode surface, current densities between 1.135 and 1.31 A cm^−2^ were selected.

### 3.2. Mass Removed During the PEP Process

**Figure 2:**
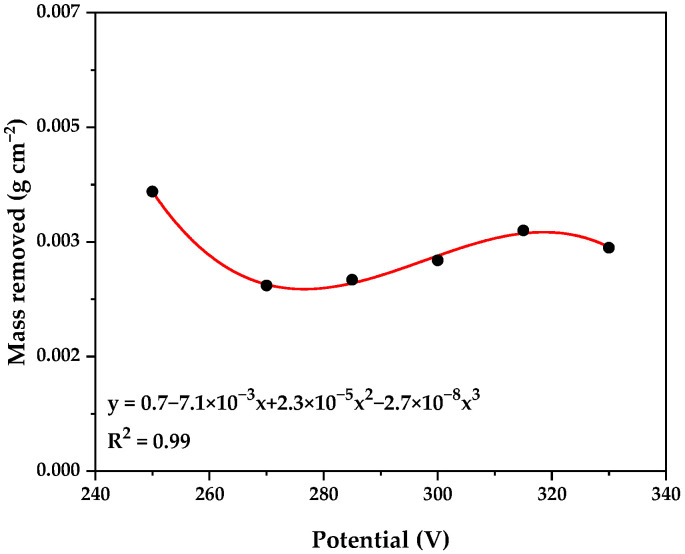
Mass removed during PEP at different potentials.

Figure 2 illustrates the average mass removed at different potentials during the Plasma Electrolytic Polishing (PEP) process. Unlike observations by Wang et al. [25] and Rajput et al. [26], who reported a proportional relationship between increased voltage and decreased roughness, our experiments showed that an increase in voltage did not significantly affect material removal. This was likely due to a capacitive effect generated by the vapor sheath, which led to a slight reduction in the amount of mass removed until a critical value, known as the breakdown value, was reached. Notably, a mirror-like surface finish was achieved in the range of 250 to 330 V.

**Figure 3:**
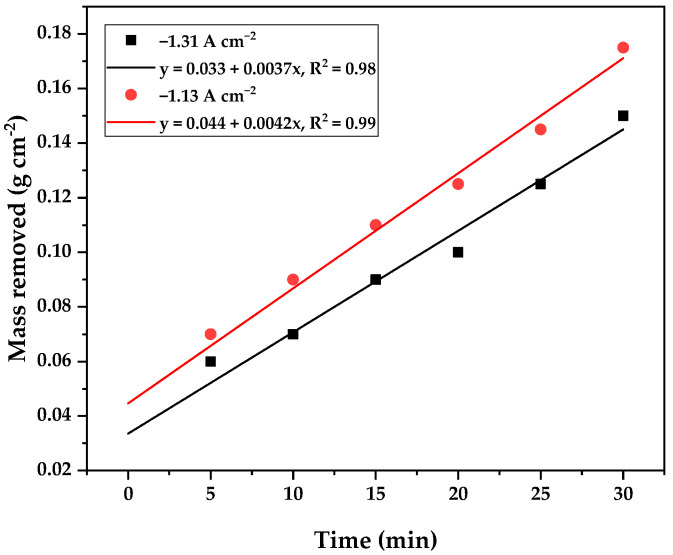
Amount of mass removed from the AISI 304 SS as a function of time. The data was obtained at a potential of 300 V, using current densities of 1.13 and 1.31 A cm^−2^.

Figure 3, on the other hand, displays the amount of mass removed from the AISI 304 SS surface at 300 V as a function of time, using current densities of 1.135 and 1.31 A cm^−2^. We observed that a greater amount of material was removed with longer times and higher current densities. This is attributed to a larger amount of energy being available for anodic dissolution reactions, as suggested by Kalenchukova et al. [22]. The average mass removed over 20 min at 300 V and a current density of 1.31 A cm^−2^ was 0.0218 g cm^−2^. However, it is important to note that increasing the current density from 1.135 A cm^−2^ to 1.31 A cm^−2^ did not yield a significant benefit in terms of the amount of material removed.

### 3.3. Surface Analysis

Roughness was evaluated on six samples polished for 20 min at 300 V under plasma conditions with a current density of 1.135 A m^−2^. Roughness results were obtained by measuring three points on the surface of each sample.

The average initial roughness of the untreated specimens was 0.197 ± 0.023 μm. In contrast, the average final roughness of the samples polished by the PEP process was 0.0895 ± 0.0205 μm. These results demonstrate a 54% reduction in average roughness, leading to a mirror-like finish.

**Figure 4:**
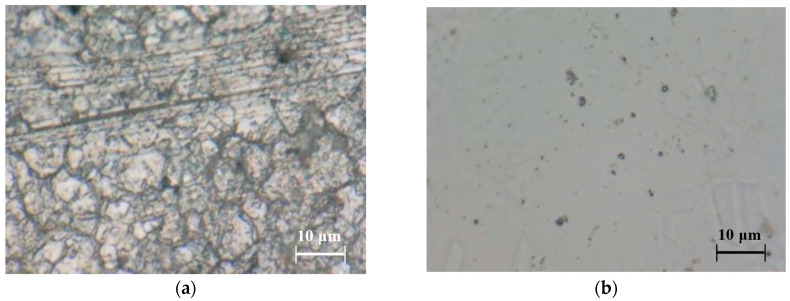
Microstructures of AISI 304 SS (4000×): (**a**) microstructure of untreated AISI 304SS steel and (**b**) microstructure of steel electropolished for 20 min at 300 V.

Figure 4 displays the stainless steel pieces before and after electropolishing under electrolytic plasma conditions. The elimination of surface imperfections in the untreated steel after polishing is evident. The presence of small craters on the electropolished samples is a consequence of pitting generation on the steel surface, associated with surface corrosion during PEP.

### 3.4. Surface Chemical Analysis by X-Ray Photoelectron Spectroscopy (XPS)

**Figure 5:**
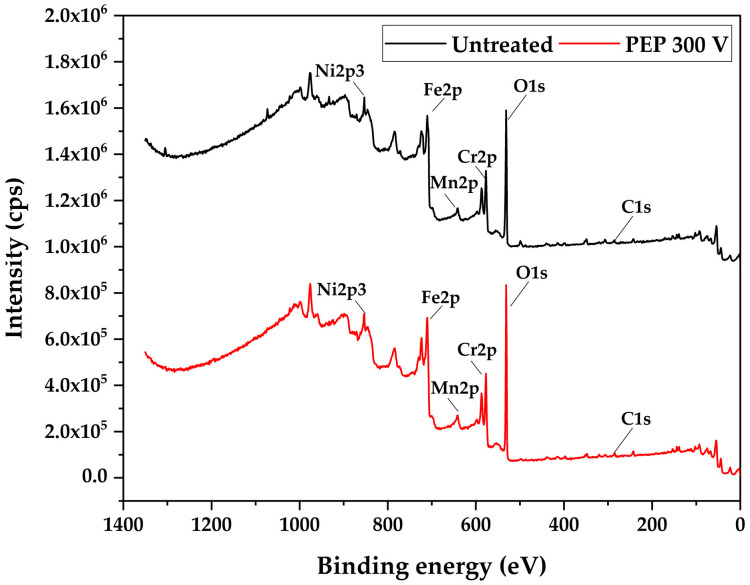
XPS survey spectra for untreated and electrodissolved AISI 304 SS surfaces.

The surface chemical modification of AISI 304 stainless steel, both before and after the electrodissolution process at 300 V, was analyzed using X-ray Photoelectron Spectroscopy (XPS). Figure 5 presents the comparative spectra of the samples in their initial (untreated) state and after the electrodissolution treatment.

The most intense peaks correspond to the electronic levels of Cr2p, Fe2p, and O1s. The latter is associated with the formation of surface oxides during the process. Additionally, the presence of C1s was detected, attributed to adventitious carbon from environmental contamination adsorbed on the surface.

The O1s peak (~530 eV) significantly increases after PEP treatment at 300 V, indicating the formation of an oxygen-rich oxide layer. This oxygen primarily originates from the electrochemical dissociation and decomposition of water molecules (H_2_O) present in the aqueous electrolyte at the anode–electrolyte interface under high-voltage and high-plasma-discharge conditions. Additionally, the ammonium nitrate (NH_4_NO_3_) in the solution may also contribute to the generation of reactive oxygen species. The presence of carbon (C1s) remains consistent in both samples, likely due to surface contamination.

Overall, these changes suggest that PEP promotes surface passivation through the formation of metallic oxides, which can improve the corrosion resistance of the treated material.

### 3.5. XPS Analysis of Cr2p3/2

**Figure 6:**
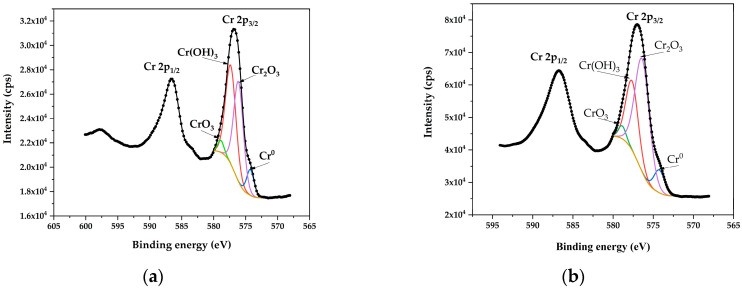
XPS spectra of chromium (Cr2p3/2) for (**a**) untreated AISI 304 SS and (**b**) AISI 304 SS treated with PEP at 300 V.

Figure 6 displays the XPS spectra of chromium (Cr2p3/2), comparing untreated steel to steel polished under PEP at 300 V. XPS analysis allowed for the determination of surface chemical characteristics through binding energies (Table 1), which can be used to identify the oxidation states of Cr, and percent chemical composition (Table 2), which quantifies the distribution of chemical species.

After electropolishing, an increase in Cr_2_O_3_ concentration and a reduction in Cr(OH)_3_ concentration were observed on the treated steel surface, compared to untreated steel. This suggests that electropolishing increases the passive oxide content on the steel surface by accelerating the transformation of hydroxide into Cr_2_O_3_, which aligns with a previously mentioned reaction [27]. This phenomenon also indicates that the transformation of Cr(OH)_3_ to Cr_2_O_3_ is a slower process, as metallic Cr must first react with water to form Cr(OH)_3_ before transforming into Cr_2_O_3_ [27].

### 3.6. XPS Analysis of Fe2p3/2

**Figure 7:**
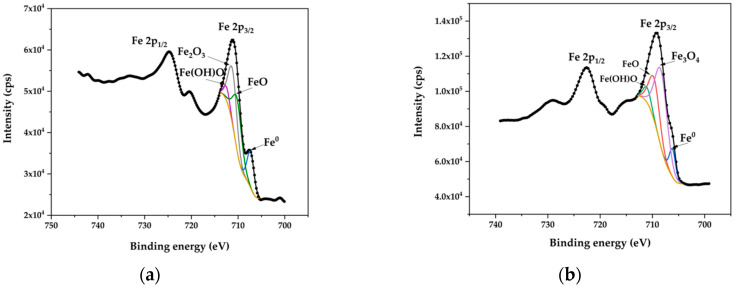
XPS spectra of iron (Fe2p3/2) for (**a**) untreated AISI 304 SS and (**b**) AISI 304 SS PEP-treated at 300 V.

Figure 7 shows the XPS spectra of iron (Fe2p3/2), comparing untreated steel to steel polished under electrolytic plasma conditions at 300 V. XPS analysis revealed the surface chemical characteristics through binding energies (Table 3), which can be used to identify the oxidation states of Fe, and percent chemical composition (Table 4), which quantifies the distribution of chemical species.

The primary change detected on the polished surfaces, compared to the untreated steel, is the presence of magnetite (Fe_3_O_4_) instead of hematite (Fe_2_O_3_). This modification imparts greater hardness and corrosion resistance to the surface treated with electrolytic plasma. The presence of magnetite has been reported in electrochemically polished 304 stainless steels [7,28,29,30]. Polishing under plasma conditions at 300 V resulted in the highest amount of surface Fe_3_O_4_.

According to studies by Rokosz et al. [30,31], the passive layer on abrasively polished samples primarily consists of Cr_2_O_3_ and Fe_2_O_3_ oxides, along with FeOOH and CrOOH hydroxides. In contrast, the passive layer of electropolished steel contains only Cr_2_O_3_ and Fe_3_O_4_.

### 3.7. Cr/Fe Ratio

The XPS data reveal that PEP treatment at 300 V significantly modifies the surface composition, increasing the metallic Cr/Fe ratio from 0.44 to 0.84 while promoting Cr_2_O_3_ as the dominant oxide (57.34 wt. % vs. 42.53 wt. % of untreated AISI 304 SS). The surface undergoes iron depletion (Fe^0^ drops from 19.52% to 10.37%) and phase reorganization, with Fe_3_O_4_ becoming the primary iron oxide (55.88 wt.%) instead of Fe_2_O_3_. These changes, particularly the Cr_2_O_3_ enrichment and metallic Cr^0^ increase, explain the enhanced corrosion resistance. This behavior has already been studied in previous works, such as that by Kerber and co-workers [32], who agree that electropolishing increases the corrosion resistance of steel in a salt chamber since it increases the Cr/Fe ratio on the surface.

### 3.8. General Corrosion

**Figure 8:**
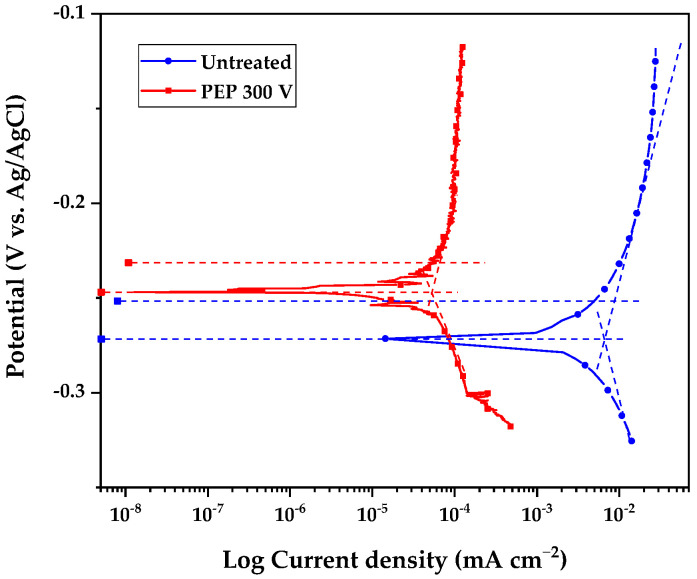
Uniform corrosion of AISI 304 SS through different surface modifications.

The corrosion analysis included the evaluation of uniform corrosion through corrosion potential (E_corr_) and corrosion current density (i_corr_) measurements using the Tafel technique and polarization resistance. For these measurements, the exposed areas were calculated for untreated 304 SS (7.67 cm^2^) and PEP-treated 304 SS (5.80 cm^2^), calculated by Equation (1). From these values, the corrosion rate (CR) was calculated. Figure 7 shows the behavior of untreated AISI 304 SS and AISI 304 SS superficially modified by PEP at 300 V. Table 5 shows the corrosion parameters obtained from the Tafel extrapolation (Figure 8) and the corrosion rate calculated from Equation (2) [23].

The electrochemical results demonstrate that PEP treatment at 300 V significantly improves the corrosion resistance of AISI 304 SS in simulated seawater, showing a nobler potential (−0.246 V vs. −0.274 V for the untreated material) and a reduction of three orders of magnitude in the corrosion current (0.01 vs. 12.66 μA cm^−2^). This translates into a 95% decrease in the corrosion rate (0.0001 vs. 0.094 mm yr^−1^), evidencing the formation of an effective passive layer.

Table 6 presents the corrosion rate calculated from polarization resistance (R_p_), obtained through electrochemical measurements following ASTM G102-23 [17]. The corrosion current density (i_corr_) was determined using Equation (6), and the corrosion rate (CR) was derived from Equation (2). The results demonstrate a significant improvement in corrosion resistance after Plasma Electrolytic Polishing (PEP) at 300 V compared to the untreated sample.

The untreated material exhibited a polarization resistance (R_p_) of 4024 Ω cm^2^, with a corrosion current density (i_corr_) of 8.30 μA cm^−2^, leading to a corrosion rate of 0.1105 mm yr^−1^. In contrast, the PEP-treated sample showed a drastically higher R_p_ (40,080 Ω cm^2^), indicating enhanced passivation. This was reflected in the much lower i_corr_ (8.6 × 10^−4^ μA cm^−2^) and a corrosion rate of just 0.0086 mm yr^−1^—a ~ 90% reduction compared to the untreated condition. The Tafel slopes (β_a_ and β_c_) remained relatively stable, suggesting that the corrosion mechanism was not significantly altered, but the greatly increased R_p_ confirmed the formation of a highly protective passive layer. These results highlight the effectiveness of PEP in improving corrosion resistance and the utility of LPR measurements in monitoring passivation dynamics.

Both electrochemical techniques conclusively demonstrate that Plasma Electrolytic Polishing (PEP) at 300 V provides exceptional corrosion protection, reducing the corrosion rate (CR) by approximately 90–95% compared to untreated samples. The observed discrepancies between Tafel extrapolation and linear polarization resistance (LPR) measurements stem from fundamental differences in their operational principles. The Tafel method, which evaluates corrosion behavior across wider polarization ranges (±100–300 mV), may be influenced by non-linear polarization effects in passive systems. In contrast, LPR measurements (typically limited to ±20 mV perturbations near the corrosion potential) are more sensitive to minor electrochemical changes under near-equilibrium conditions [33]. For PEP-treated materials, this distinction becomes particularly relevant: Tafel analysis reflects the material’s performance under aggressive polarization (demonstrating superior protection), while LPR captures subtle film reorganization processes that occur under steady-state conditions. These complementary perspectives provide a more comprehensive understanding of the passive film’s protective mechanisms.

### 3.9. Pitting Corrosion

**Figure 9:**
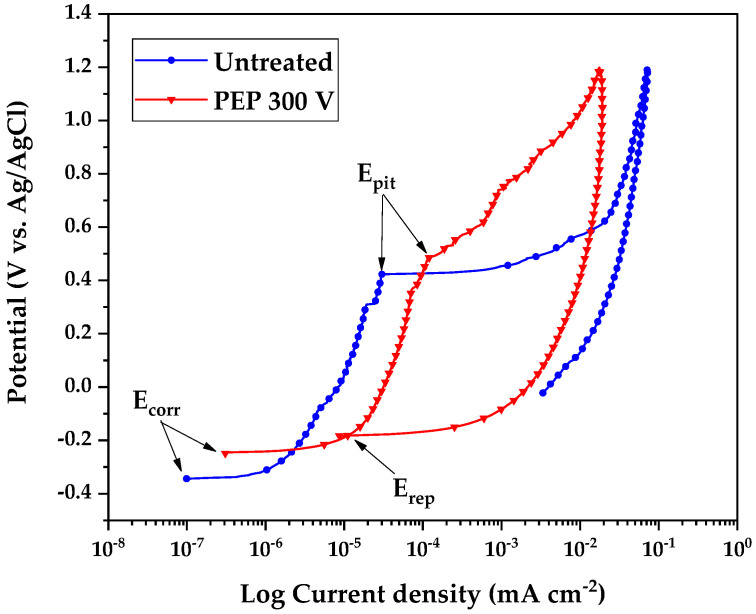
Anodic branch of the polarization curves applied to untreated 304 stainless steel (blue line) and 304 stainless steel treated with PEP at 300 (red line).

The localized corrosion analysis (Figure 9) and electrochemical parameters (Table 7) show that AISI 304 SS treated with PEP at 300 V exhibits significantly improved behavior, evidenced by its nobler pitting potential (E_pit_ = 0.486 V vs. 0.423 V for the raw material), its corrosion potential being shifted to more positive values (−0.245 V vs. −0.344 V), and the appearance of repassivation capacity (E_rep_ = −0.191 V), which was absent in the untreated material. This improvement is attributed to the surface modification induced by PEP, which promotes the formation of a more stable and thicker passive layer, an effect enhanced by the adsorption of nitrate ions, which inhibit the nucleation of pits until the transpassivity regime is reached, as reported by Calderón et al. [34] and Seyedi et al. [35].

The anticorrosive effect on samples polished under plasma conditions is associated with an increase in the Cr/Fe ratio in their metal elements, which means a high possibility of further increasing the formation of protective oxides due to the highly spontaneous tendency of chromium to form oxides with respect to iron. In addition, the oxides formed on the surface include Fe_3_O_4_ and Cr_2_O_3_.

These phenomena have been observed by Mandrino et al. [36] and Rokosz et al. [30,31] in different stainless steels electropolished under various conditions. They observed the presence of Fe_3_O_4_ and the oxides FeO and Fe_2_O_3_ in the electropolishing of high-current-density SS, which indicates that FeO species can be considered an intermediate phase between Fe^0^ and Fe_2_O_3_ species. Under the conditions of our research, all Fe_2_O_3_ was replaced by Fe_3_O_4_. This suggests that in these unique electrolyte plasma conditions, sufficient energy is obtained to promote the direct oxidation of Fe^0^ into a stable, mixed-valence iron oxide layer rich in Fe^2+^ and Fe^3+^ species, leading to the preferential formation of Fe_3_O_4_. This is formed as an oxidation product inherent to the high-electric-field and high-temperature conditions present at the anode during the PEP process, rather than from a reduction process.

During the Plasma Electrolytic Polishing (PEP) process, the predominant anodic reactions, such as metal dissolution and water oxidation, lead to the localized generation of H+ ions at the electrode surface. This results in a tendency for the solution to acidify near the anode. This consideration is important since plasma conditions make direct pH measurement difficult. The Fe-O-H diagram shows that, at pH values below 5 and at potentials above 0.75 V, the predominant species are Fe^3+^, FeOH^2+^, and FeO^+^. These conditions promote the oxidation of Fe^0^ on the surface. The XPS spectra, which show the presence of Fe_3_O_4_, FeO, and FeOOH without the presence of hematite (Fe_2_O_3_), suggest that under the high-energy, non-equilibrium conditions characteristic of electrolytic plasma, direct oxidation favors the formation of these mixed-valent and lower-valent iron oxide phases as part of the passive layer.

This same analysis can be applied to chromium species. According to the Pourbaix diagram of the Cr-O-H system [37], it can be observed that the predominant species at pH values less than 5 are Cr^3+^ and HCrO^4–^, which correspond to the species observed in the XPS spectra, where the presence of Cr(OH)_3_ and Cr_2_O_3_ is detected, of which only Cr_2_O_3_ grows in concentration during PEP. This implies the growth of the protective passive layer during the polishing treatment. Interestingly, plasma conditions do not modify Cr^6+^ (CrO_3_) species, where no substantial change in chromic oxide was noted.

## 4. Conclusions

Electrolytic Plasma Polishing (PEP) at 300 V, using an aqueous solution of urea (3.0 wt.%) and NH_4_NO_3_ (0.25 wt.%), achieved a remarkable reduction in the surface roughness of untreated AISI 304 SS. The surface roughness decreased by 54.6% (from 0.197 ± 0.023 μm to 0.0895 ± 0.0205 μm) with minimal mass loss (0.0035 g·cm^−2^) in just 20 min.

Tafel analysis showed a 99% reduction in the corrosion rate (0.0086 mm yr^−1^) compared to untreated AISI 304 SS (0.11 mm yr^−1^). Cyclic Potentiodynamic Polarization (CPDP) measurements indicated superior pitting resistance (E_pit_ = +0.423 vs. +0.486 V for PEP processing). XPS analysis elucidated the underlying mechanisms, showing a 91% increase in the Cr/Fe ratio (0.44 to 0.84) and complete transformation of surface oxides to protective Cr_2_O_3_ (57.34 wt.%) and Fe_3_O_4_ (55.88 wt.%), which collectively explain the enhanced corrosion resistance.

## Figures and Tables

**Table 1:** Core-level binding energies (eV) of Cr compounds before and after Plasma Electrolytic Polishing (PEP) at 300 V.

Compound	Binding Energy, eV	
	Untreated	PEP 300 V
**Cr^0^**	574.27	574.24
**Cr_2_O_3_**	576.11	576.37
**Cr(OH)_3_**	577.34	577.64
**CrO_3_**	578.88	578.87

**Table 2:** Chemical composition of surface chromium species: untreated vs. PEP-treated at 300 V.

Compound	Composition, wt. %	
	Untreated	PEP 300 V
**Cr^0^**	8.57	8.66
**Cr_2_O_3_**	42.53	57.34
**Cr(OH)_3_**	45.5	30.5
**CrO_3_**	3.4	3.5

**Table 3:** Core-level binding energies (eV) of Fe compounds before and after Plasma Electrolytic Polishing (PEP) at 300 V.

Compound	Binding Energy, eV	
	Untreated	PEP 300 V
Fe^0^	706.91	706.16
Fe_3_O_4_	-	708.32
FeO	709.72	709.63
Fe_2_O_3_	710.77	-
Fe(OH)O	712.05	711.02

**Table 4:** Chemical composition of surface iron species: untreated vs. PEP-treated at 300 V.

Compound	Composition, wt. %	
	Untreated	PEP 300 V
Fe^0^	19.52	10.37
Fe_3_O_4_	-	55.88
FeO	37.11	26.81
Fe_2_O_3_	37.08	-
Fe(OH)O	6.29	6.94

**Table 5:** Corrosion behavior in simulated seawater of different AISI 304 SS surfaces.

Treatment	E, V	i_corr_, μA cm^−2^	CR, mm yr^−1^
Untreated	−0.274	12.66	0.125
PEP 300 V	−0.246	0.0101	0.0001

**Table 6:** General corrosion parameters determined using the polarization resistance (R_p_) technique.

Treatment	∆E, V	I_med_, A cm^−2^	R_p_, Ω cm^2^	β_a_, V dec^−1^	β_c_, V dec^−1^	B, mV	I_corr_, μA cm^−2^	CR, mm yr^−1^
Untreated	0.02	6.61 × 10^−6^	4024.14	0.213	0.121	33.64	8.30	0.1105
PEP 300 V	0.02	5.04 × 10^−7^	40080.16	0.192	0.133	34.25	0.0086	0.0086

**Table 7:** Pitting corrosion curves recorded by CPDP at 0.6 V h^−1^ in simulated seawater for untreated and electropolished AISI 304 SS surfaces.

Treatment	E_corr_, V	E_pit_, V	R_pit_, V	E_rep_, V
Untreated	−0.344	0.423	0.767	-
PEP 300	−0.245	0.486	0.731	−0.191

## Data Availability

The original contributions presented in the study are included in the article material, and further inquiries can be directed to the corresponding authors.

## Abbreviations

The following abbreviations are used in this manuscript:

CPP	Cyclic Potentiodynamic Polarization
CR	Corrosion Rate
PEP	Plasma Electrolytic Polishing
R_p_	Polarization Resistance
R_pit_	Pitting Resistance
SS	Stainless Steel
